# An Integrative Way for Studying Neural Basis of Basic Emotions With fMRI

**DOI:** 10.3389/fnins.2019.00628

**Published:** 2019-06-19

**Authors:** Simeng Gu, Fushun Wang, Caiyun Cao, Erxi Wu, Yi-Yuan Tang, Jason H. Huang

**Affiliations:** ^1^Institute of Brain and Psychological Science, Sichuan Normal University, Chengdu, China; ^2^Department of Psychology, Jiangsu University, Zhenjiang, China; ^3^Department of Pharmacology, Nanjing University of Chinese Medicine, Nanjing, China; ^4^Department of Neurosurgery, Baylor Scott & White Health, Temple, TX, United States; ^5^Department of Surgery, Texas A&M University College of Medicine, Temple, TX, United States; ^6^Department of Pharmaceutical Sciences, Texas A&M University College of Pharmacy, College Station, TX, United States; ^7^LIVESTRONG Cancer Institutes, Dell Medical School, The University of Texas at Austin, Austin, TX, United States; ^8^Department of Psychological Sciences, Texas Tech University, Lubbock, TX, United States

**Keywords:** basic emotions, core affects, monoamine, dimensional studies, fMRI

## Abstract

How emotions are represented in the nervous system is a crucial unsolved problem in the affective neuroscience. Many studies are striving to find the localization of basic emotions in the brain but failed. Thus, many psychologists suspect the specific neural loci for basic emotions, but instead, some proposed that there are specific neural structures for the core affects, such as arousal and hedonic value. The reason for this widespread difference might be that basic emotions used previously can be further divided into more “basic” emotions. Here we review brain imaging data and neuropsychological data, and try to address this question with an integrative model. In this model, we argue that basic emotions are not contrary to the dimensional studies of emotions (core affects). We propose that basic emotion should locate on the axis in the dimensions of emotion, and only represent one typical core affect (arousal or valence). Therefore, we propose four basic emotions: joy-on positive axis of hedonic dimension, sadness-on negative axis of hedonic dimension, fear, and anger-on the top of vertical dimensions. This new model about basic emotions and construction model of emotions is promising to improve and reformulate neurobiological models of basic emotions.

## Introduction

Emotion is a kind of mental state that occurs at almost all times across life. Despite the critical importance of emotions in our lives, there is currently no scientific consensus on a definition about what emotion is (Gu et al., 2015). Controversy still abounds over the definition of emotions; the number of emotions that exit. Fehr and Russell (1984) wrote that “everyone knows what an emotions is, until asked to give a definition. Then, it seems no one knows” (Fehr and Russell, 1984). Emotions are internal states that are evoked by comparison between the internal bodily needs and the available external materials, and are characterized by induced physiological changes, behavioral and cognitive changes (Sroufe, 1996; Wang, 2018). The James-Lange Theory of Emotion suggested that the perception of these bodily changes is what is called an emotion. However, emotions themselves are neither physiological changes nor behavioral changes, because emotions can be separated from behavioral changes, for example, you can hold back fighting behaviors even though you are angry (Damasio and Carvalho, 2013; Wang, 2018). Anyway, emotions can only be consciously known by human beings, and only human beings can consciously regulate their emotions (Etkin et al., 2015, 2016; Hay et al., 2015; Milyavsky et al., 2018); animals cannot hold back the emotion related physiological changes or behaviors. Therefore, it is hard to say emotions are only neuromodulators separately or inseparately with behaviors. However, emotions can only be studied with these external expressions, such as physiological or behavioral changes, especially facial expressions, because we still cannot study animal emotions directly as we have no access to an organism’s subjective experience (Papini et al., 2018). Darwin was the first to use facial expressions in emotional studies, such as fear, anger, joy, or sadness. Later, behaviorists tried to study behaviors using emotion induced physiological changes, such as saliva in Palov’s dog, and other behavior changes by Skinner’s pigeon at a reward or punishment. All these studies did not answer one critical question about emotions: How many kinds of emotions humans have. In contrast, the question is getting more emergent.

During the past decades, basic emotion theory has been very influential in the field of affective studies (Saarimaki et al., 2016; Celeghin et al., 2017; Williams, 2017; Hutto et al., 2018; Song and Hakoda, 2018; Vetter et al., 2018; Wang et al., 2018), which proposed that all human emotions are composed of limited number of basic emotions (e.g., fear, anger, joy, sadness), which are considered to be more elementary than others. These emotions are called basic emotions, for they are biologically and psychologically “basic”. These basic emotions are related to our basically biological needs (Lindquist and Barrett, 2012; Colombetti, 2014; An et al., 2017), and each emotion has its own dedicated neural circuitry that is architecturally distinct (Ekman, 1992; Russell, 2006; Barrett and Russell, 2015; Scarantino, 2015). For example, Izard argued that the basic emotions are preserved because their biological and social functions are essential in evolution and adaption, and he suggested that basic emotions have innate neural substrates and universal behavioral phenotypes (Damasio and Carvalho, 2013). Even though many psychologists accepted the theory of basic emotion, there is no consensus as to the exact number of basic emotions. For example, Ekman proposed six basic emotions: happiness, anger, sadness, fear, disgust, and surprise, while Izard proposed seven basic emotions: fear, anger, happiness, sadness, disgust, interest, and contempt. In addition, several recent papers, depending on facial expression studies and neural mechanism studies, suggest four basic emotions: fear, anger, joy, and sadness (Jack et al., 2014; Gu et al., 2016, 2018; Zheng et al., 2016). Many other psychologists also proposed many other basic emotions, as reviewed in a paper by Ortony in *Emotion Review* (Clore and Ortony, 2013), shown in the Table 1.

**TABLE 1:** Basic emotions.

**Theorist**	**Basic emotions**
Arnold	Fear, anger, desire, despair, hope, love, courage, sadness, aversion, hate, dejection
Plutchik	Fear, anger, joy, sadness, anticipation, disgust, surprise, acceptance
Ekman, Friesen, and Ellsworth	**Fear, anger, joy, sadness, disgust, surprise**
Frijda	Joy, surprise, wonder, sorrow, interest, desire
McDougall	Fear, anger, disgust, wonder, elation, subjection, tender-emotion
Izard	Fear, anger, joy, contempt, disgust, distress, shame, guilt, interest, surprise
Tomkins	Fear, anger, joy, interest, contempt, disgust, distress, shame, surprise
Oatley and Johnson-Laird	Anger, happiness, sadness, anxiety, disgust
Gray	Rage and terror, anxiety, joy
Fushun Wang, and Jack RE	Fear, anger, joy, sadness

The basic emotion theory proposed that each emotion has its own dedicated neural circuitry that is architecturally distinct. For example, fear is a kind of emotion that produce subjective feelings through separate neural pathways of the central nervous system, or peripheral nervous systems (Cowen and Keltner, 2018). Therefore, studying the neural basis might be the best way to differentiate the basic emotions and probe into the number of basic emotions. However, many fMRI studies have met some troubles in differentiating the basic emotions (such as fear, anger, joy, sad, and disgust) with distinct universal signals, physiology, especially the localization of the central nervous system (Lindquist and Barrett, 2012; Lindquist et al., 2012), which has led to even more controversies about the basic emotions (Posner et al., 2005; Scarantino and Griffiths, 2011; Scarantino, 2015; Hutto et al., 2018). For example, even though neuroimaging studies found some evidence for basic emotions [such as amygdala for fear (Ohman, 2005), insula for disgust (Wicker et al., 2003), anterior cingulate cortex for sadness, orbitofrontal cortex for anger (Murphy et al., 2003)], these neuroimaging data are not consistent with specific one-to-one correspondence between fMRI localization of anger, sadness, fear, disgust, etc (Lindquist et al., 2012; Clark-Polner et al., 2017). These neuroimaging data have challenged the basic emotion theory (Hamann, 2012; Scarantino, 2012; Barrett and Satpute, 2013; Cowen and Keltner, 2018), leading many psychologists to suspect (if not give up) the basic emotion theory (Scarantino and Griffiths, 2011; Colombetti, 2014; Scarantino, 2015; Cowen and Keltner, 2018; Hutto et al., 2018). Actually, the reason for the complication might be due to the fact that the basic emotions used in these experiments are not “basic” enough, as they can be further divided into even more “basic” emotions; or the “basic emotions” used in previous reports did not in fact represent the most psychologically primitive levels of emotion (Lindquist and Barrett, 2012). Indeed, Colombetti (2014, p. 38) suggested that the six basic emotions in Ekman’s study were not chosen “on the basis of a clear rationale” (Colombetti, 2014). Indeed, they chose the six emotions because they could not obtain enough suitable sample photographs (Colmbetti, 2014; Hutto et al., 2018). Here we probe into the question whether humans have four basic emotions, based on fMRI data.

## fMRI Data on Basic Emotions

Recent studies with fMRI offer a good opportunity to study the underlying brain mechanisms for basic emotions, and these neuroimaging studies found some specific loci in the brain work for basic emotions, while other regions are generally involved in emotion perception, valuation, or regulation (Phan et al., 2002; Lindquist et al., 2012, 2013b).

### Happiness

Happiness is one of the human resources that an individual can pursuit in his life. The psychological study of happiness defined two different conceptions of happiness: hedonic happiness and eudaimonic happiness (Berridge and Kringelbach, 2011). Hedonic happiness is what we feel during the experience of intense physical or psychological pleasure, while eudaimonic happiness is what we feel when we reach our personal goals or when we have expressed our potential, our abilities, or to be who we really are (Berridge and Kringelbach, 2011). Indeed, imagination of happy events from both kinds, compared to the imagination of neutral events, activates the ventral prefrontal cortex (including orbitofrontal cortex) (Kringelbach, 2005). In a functional magnetic resonance imaging (fMRI) experiment, Rolls et al. (2008) found that activations in the ventral prefrontal cortex, the cingulate cortex, and the ventral striatum were associated with the positive hedonic state, depending on the correlations between the ratings of the participants on pleasantness with the blood-oxygen-level dependent (BOLD) signal. Later on, many studies provide evidence for the primordial role played by these areas in hedonic valuation (Rolls et al., 2008; Grabenhorst and Rolls, 2011). There is almost no controversy regarding the involvement of the ventromedial prefrontal cortex in the subjective happiness and hedonic value (Abler et al., 2005). However, it is still uncertain whether these frontal regions can cause pleasure, and data from lobotomised patients do not indicate a total loss of pleasure; on the contrary, some patients showed euphoria, impulsiveness and general disinhibition. Similarly, Beer et al. (2003) demonstrated the performance of good humor and self-satisfaction in patients with orbitofrontal damage. *These data suggest that the OFC could be more important in transforming pleasure stimuli into their cognitive representations* (Burke et al., 2007, 2008, 2009; Takahashi et al., 2009). In addition, there are some reports about the differences between hedonic and eudaimonic happiness; where hedonic pleasure was positively correlated with functional connectivity of bilateral ventral medial prefrontal cortex, while eudaimonic pleasure was shown to be related to bilateral precuneus (Luo et al., 2017). In addition, it is found that ventral striatum activation during eudaimonic decisions predicted longitudinal declines in depressive symptoms, whereas, ventral striatum activation to hedonic decisions were related to longitudinal increases in depressive symptoms (Telzer et al., 2014).

The mesolimbic dopaminergic system has been considered to be able to cause pleasure (Yokel and Wise, 1975; Wise and Rompre, 1989; Wise, 2004, 2006, 2008, 2013). Even though it is hard to visualize the ventral tegmental area (VTA) with fMRI, due to its lacking clear anatomical borders (Trutti et al., 2019), there are some fMRI studies that report the activities of VTA in the reward and happiness (Krebs et al., 2011). In addition, some reports suggest that VTA responses correlated with romantic love scores and inclusion of other in the self (Acevedo et al., 2012; Xu et al., 2012). VTA is the origin of the mesolimbic dopaminergic system, which projects and releases dopamine to the locus coeruleus, prefrontal cortex and anterior cingulate cortex, and it is responsible for the cognitive effects of positive emotion.

### Sadness

Sadness is an emotion that is indicative of loss and helplessness (Motoki and Sugiura, 2018), or it is related to failure to get wanted thing (reward), or punishment to get harmful things (Gu et al., 2016). Anterior cingulate cortex (ACC) is related to sadness (Godlewska et al., 2018; Ramirez-Mahaluf et al., 2018a, 2018b). The reason for ACC to be responsible for sadness might be due to the fact that it is the brain side to induce the vocalization for crying response, which is supported by neuroimaging studies. In addition, it is also linked to sadness because of its role in suffering; many studies have suggested ACC is also implicated in pain or the suffering feeling and depression (Taylor et al., 2018). Therefore, ACC is supposed to be the location for frustration, punishment, regret or failure to cope with the situation (Abler et al., 2005). Lateral orbitofrontal area is also involved in unpleasant stimuli, and it is reported that activations in the lateral parts of the orbitofrontal cortex were related to the negative hedonic value.

### Fear

The amygdala is an important limbic structure that has been associated with fear (Anthony et al., 2014; Isosaka et al., 2015; Reynaud et al., 2015; Han et al., 2017). Many fMRI studies support the hypothesis that amygdala is the most important hub in a fear reaction (LeDoux, 1998). Several aspects of fear processing have been attributed to the amygdala, including fear conditioning (Davis, 1992; LeDoux, 2007), initiation of fear-induced behaviors in response to stressors (Weiskrantz, 1956; Blanchard and Blanchard, 1972; Prather et al., 2001; Izquierdo et al., 2005; Machado et al., 2009), and memory creation of fear-related stimuli (Cahill et al., 1995; Hamann, 2001). The importance of the amygdala in initiating the fear emotion and fear-related behaviors has been well described by studying amygdala damage in non-human animals, in which decreased fear-related behaviors have been observed with poor amygdala function (Weiskrantz, 1956; Blanchard and Blanchard, 1972; Prather et al., 2001; Izquierdo et al., 2005; Machado et al., 2009). In addition, clinical observations of a well-characterized human case of bilateral amygdala damage confirmed that amygdala is the location of fear (Feinstein et al., 2011). However, many studies have found that amygdala is involved with many other negative emotions too, such as stress or anger (Siep et al., 2018), which might be due to the emotion flow: fear emotion is transient and can induce several other emotions, such as anger (Zheng et al., 2016). Animals under the influence of fear try to flight away from the threat, however, they will defend themselves, usually aggressively, when flight is impossible or difficult (Papini et al., 2018). This kind of “fight or flight” behavior or “fear and anger” emotion usually happens interchangeably (Zheng et al., 2016).

### Anger

Orbitofrontal cortex is the location for anger, because of its relation to prey. Many fMRI studies suggest that the interaction between orbitofrontal cortex and amygdala is involved in the regulation of anger (Coccaro et al., 2007; Fulwiler et al., 2012). It is reported that hyperactivity of the interaction between amygdala and orbitofrontal cortex is involved in the altered fear/anger processing at stressful situations (Reynaud et al., 2015). However, some findings also show a hypoactivation in the orbitofrontal cortex (OFC) and the anterior cingulate cortex (ACC), but strong activity in amygdala (Fulwiler et al., 2012; Siep et al., 2018). A meta-analysis study confirmed enhanced activation in the left amygdala to angry stimuli (Bertsch et al., 2018; Krauch et al., 2018). These controversies might be due to the fact that amygdala activity is related to initiation of fear, while activation of the orbitofrontal cortex is involved in the fear extinction (Milad and Rauch, 2007; Siep et al., 2018). Fear extinction is sure to induce anger, which confirmed a previous report that “fight or flight” behavior or “fear and anger” emotion usually happens interchangeably, or that anger is the vent for fear and fear leads to anger (Gu et al., 2016).

### Disgust

The emotion of disgust is typically experienced as a feeling of revulsion elicited by offensive stimulations – e.g., bodily fluids and waste, animal products, rotten food, and certain classes of sexual behavior (such as incest), and is accompanied by a strong desire to throw the eliciting stimulus away (Oaten et al., 2018a, b). Although the **insula** has been proposed as the seat of disgust processing, which is considered to be distinguishable from other emotive responses – e.g., fear (Wicker et al., 2003), and anger (Wicker et al., 2003; Felmingham et al., 2008; Williams and Bargh, 2008; Koritnik et al., 2009), meta-analysis of imaging data found that the anterior insula is not more active during disgust than other emotions, such as anger (Wager et al., 2007; Oaten et al., 2018a, b). Indeed, disgust in moral situations often induces anger, which suggests that any investigation of the neural correlates of disgust also include the anger related neural network. This is also supported by past studies that report the words “disgust” and “disgusted” evoke feelings associated with anger-related concepts (Oaten et al., 2018a, b), and this is consistent with the report that disgust and anger might be the same kind of basic emotion (Jack et al., 2014).

### Surprise

The surprise emotion alerts the individual of any deviations from expectations, regardless of the outcome value (Litt et al., 2011; Fouragnan et al., 2018). The surprise system works as an attentional mechanism that enables an organism to focus its limited energy on the most salient stimuli (Matsumoto and Hikosaka, 2009; Kahnt et al., 2010; Park et al., 2010). In addition, surprise system can also monitor unexpected information and help plan appropriate behavioral adjustments (Fouragnan et al., 2018). According to meta-analyses of fMRI studies, surprise induced brain regions are predominantly subcortical, including the amygdala and striatum, as well as some cortical regions, such as the ventromedial prefrontal cortex and the cingulate cortex (Behrens et al., 2009; Bartra et al., 2013). This is consistent with the accumulated imaging evidence, which suggest that the amygdala plays a key role in the processing of novel stimuli (Blackford et al., 2010), and also that surprise and fear might be the same basic emotions (Jack et al., 2014).

In all, many imaging data have reported specific loci for specific emotions, such as amygdala for fear (Ohman, 2005), ventromedial frontal cortex for happiness, anterior cingulate cortex for sadness, orbitofrontal cortex for anger (Murphy et al., 2003), insula for disgust (Wicker et al., 2003). Similarly, to previous reports that anger and disgust induce similar facial expressions, “disgust” and “disgusted” evoke feelings associated with anger-related concepts, and both disgust and anger can activate insula. Similarly, fear and surprise also activate similar brain loci, such as amygdala, and surprise can induce ventromedial prefrontal cortex and the cingulate cortex, because happiness can also be involved with surprise.

## Two Dimensions of Basic Emotions

Even though imaging data support the four basic emotions, *basic emotions are transient and usually interchangeable, and it might be easy to get confusing data from fMRI studies* (Lindquist et al., 2013a), for example, fear and anger emotion usually activate amygdala. Therefore, many people suspected distinct brain regions for the perception of different emotion categories, and hypothesized that it might be easier to find unique neural signatures for the core affects, such as valence and arousal (Wilson-Mendenhall et al., 2013; Bestelmeyer et al., 2017). All emotions are constructed by core affects, including hedonic (pleasure–displeasure) and arousal (rest-activated) (Russell, 2003; Gu et al., 2016), which constitute two independent dimensions of a quadrant, where all emotions can find their locations. Different location of each emotion on the quadrant reflects varying amounts of hedonic and arousal properties (Posner et al., 2005; Colibazzi et al., 2010). The horizontal dimension means valence (Watson and Clark, 1988), hedonic dimension (Lang et al., 1993), hedonic tone, liking and other identical items (Gu et al., 2015). The vertical dimension addresses tension and energy (Thayer, 1989), which is due to the unexpected way something happened, and is related to the arousal state of the body, so it is called arousal dimension (Zheng et al., 2016). These two dimensions are typical features of emotions, therefore they can be named as core affects (Sieger et al., 2015). Core affect is a term used to describe the feelings of hedonic pleasure and displeasure with some degree of arousal (Kuppens et al., 2013; Barrett and Russell, 2015); or core affect is the neurophysiological state consciously accessible as the simplest raw feelings, which cannot be reduced to anything simpler (Yik et al., 2011). Izard suggested that “core affect” itself is not a mental state of emotion, merely a feature of emotion (Izard, 2009).

**FIGURE 1:**
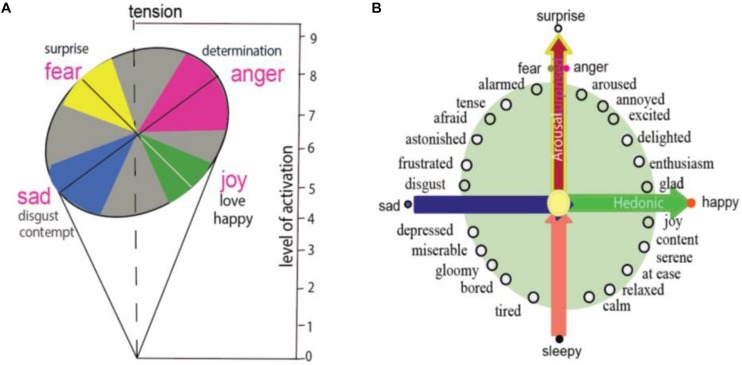
Basic emotion and circumplex. **(A)** Schlosberg proposed two-dimension of facial expression in a roughly circular surface, whose axes are pleasantness–unpleasantness and attention–rejection. The four basic emotions have different levels of activation, for example, fear and anger can reach higher levels of activation. **(B)** Circumplex model of emotion proposed that all emotions locate specially on a circle of the circumplex, means that different emotions have different *arousal or hedonic parameters*. Two core affects of emotions, which are represented on the horizontal dimension and vertical dimension, are induced by two features of a stimulus: the *safety value of the stimulus and the hedonic value of the stimulus.*

At the dawn of emotional studies, Wilhelm Wundt proposed the three-dimensional theory of emotions from the perspective of the construction approach, and suggested that emotions are caused by a set of basic common elements (Lindquist and Barrett, 2012). The three-dimensional theory postulated that human emotions result from fusion of a mixture of six basic forms of feelings: pleasure-displeasure, excitement-inhibition, tension-relaxation. Later, Schlosberg (1952) used nine-point rating scales for the tense dimension, trying to obtain independent ratings on a large number of posed facial expressions, and found that it might be better to stabilize the ratings and locate each emotion on a roughly circular surface defined by two dimensions (Schlosberg, 1954). He proposed that the whole range of facial expressions may be described in two-dimension of a roughly circular surface, whose axes are *pleasantness-unpleasantness* and *attention-rejection*. He proposed that the four emotions representing the polar of the four axes are related to activation, for example: fear and anger can reach higher levels of activation (Figure 1A), which can be compared to the primary colors as “blue-yellow and red-green axes”.

Later on, many names have been given to the two dimensions, for example, the circumplex (Figure 1B). The circumplex proposed that every emotion can be spatially represented in a circular arrangement (Posner et al., 2005; Yik et al., 2011), which is anchored on a quadrant with hedonic dimension and arousal dimensions (Kuppens et al., 2013). Barrett proposed that arousal is related to surprise or uncertainty about whether a stimulus will induce threat or reward (Gu et al., 2018). The function of arousal is the rapid detection of potential threats and can initiate appropriate approach/avoidance behaviors (fight or flight), as well as sympathetic nervous induced somatic reactions (Colibazzi et al., 2010; Lindquist et al., 2012).

### Arousal

Many psychological constructionists hypothesized that the amygdala is the location for arousal. The amygdala is most likely to be active at surprising situations, which can induce the activity of sympathetic nervous system. In addition, the amygdala has also been found to be activated at many kinds of stimulations, such as novel stimuli (Blackford et al., 2010, 2011; Weierich et al., 2010; Moriguchi et al., 2011), unusual stimuli (Blackford et al., 2011), uncertain stimuli (Herry et al., 2007), or surprise (Holland and Gallagher, 2006; Lee et al., 2006, 2008; Iordanova, 2010; Boll et al., 2013; Vrticka et al., 2014; Kim et al., 2017). Therefore, amygdala activation is not only related to fear, it is related to many highly arousing emotions, such as fear, anger, disgust (Lindquist et al., 2012). Thus, *amygdala might be the location for core affect arousal, which is related to uncertain or surprising stimuli*.

### Valence

Neuroimaging data support the idea that arousal and valence are encoded separately in the brain (Morrens, 2014). This proposition was developed on the basis of neurophysiological evidence showing that different types of neurons exhibit differential activity in response to reward or surprise (Schultz et al., 1997; Fiorillo, 2013; Fiorillo et al., 2013a, b). A stimulus that evokes emotions has two qualities: its valence (liked or disliked) and its surprise (unexpected or expected). The valence promotes an individual to reinforce an approach/ avoidance behavior, while the arousal component determines the strength of the approach/avoidance behavior (Bartra et al., 2013; Lohani et al., 2017).

## Integrative Model for Basic Emotions Approach and Construction Approach

**FIGURE 2:**
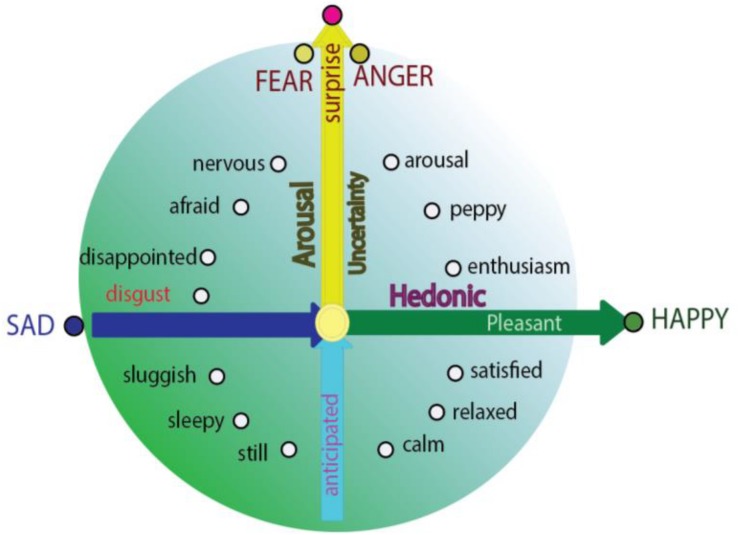
Integrative model for basic emotions and construction approach (dimension theory). Integrative model of emotion proposed that the basic emotions are on the specific locations in the circle of the circumplex; and they are typical emotions which have only one features of core affects: Fear and anger are only related to *the safety value of the stimulus*, while sadness and joy are only related to *the hedonic value of a stimulus; or* the “basic emotion” fear and anger have no hedonic value and happiness and sadness have no safety value (Zheng et al., 2016).

Even though basic emotion theory and dimensional theory have been highly influential in the field of affective studies (Hutto et al., 2018), basic emotion and dimensional theory (or called construction approach) has recently been debated (Lindquist et al., 2013b). The construction approach proposes that all the emotions are constructed from more basic “ingredients” – core affects, characterized as valence and arousal (Wilson-Mendenhall et al., 2013), and emotions are grounded in continuous and fluctuating affective states described as pleasant or unpleasant, with some level of arousal. Actually, basic emotions and dimensional studies are not contrary to each other, instead they can help make each other even clearer. Here we propose that the locations of the basic emotions are also constructed by these basic “ingredients,” and can also find their locations on the circumplex. The reason for them to be basic is because they locate specially on the circumplex, which indicates the special relationship between the basic emotions and core affects. *Fear and anger are on the vertical axis, reflecting the surprising way the stimulus happens* (Figure 2; Zheng et al., 2016); *while joy and sadness are on the horizontal dimension, depending on the hedonic value of a stimulus*. The special locations of these four basic emotions on the dimensions are the reasons why these basic emotions are “basic” (Gu et al., 2016). The specific location on the axis means that they have the highest values of dimension they locate, and it also means that they are not related to the other axis. In addition, *fear and anger have no hedonic value and happiness and sadness have no safety value* (Gu et al., 2016; Zheng et al., 2016). Of note, the basic emotions can easily interact with each other to make other emotions; for example, surprise and happiness can induce enthusiasm (Hu, 2016); or surprise and sadness might induce frustration (Figure 2; Arnott and Elwood, 2009). This is why Hu Hailan gives the equation: Happiness = reward happened – expectation (Hu, 2016). This means the basic emotions used in the experiments might not be so “basic.” Thus, we introduce a prerequisite for basic emotions: *Basic emotions should locate on the axis of the two emotional dimensions*.

### Horizontal Dimension-Direction of Behaviors

**FIGURE 3:**
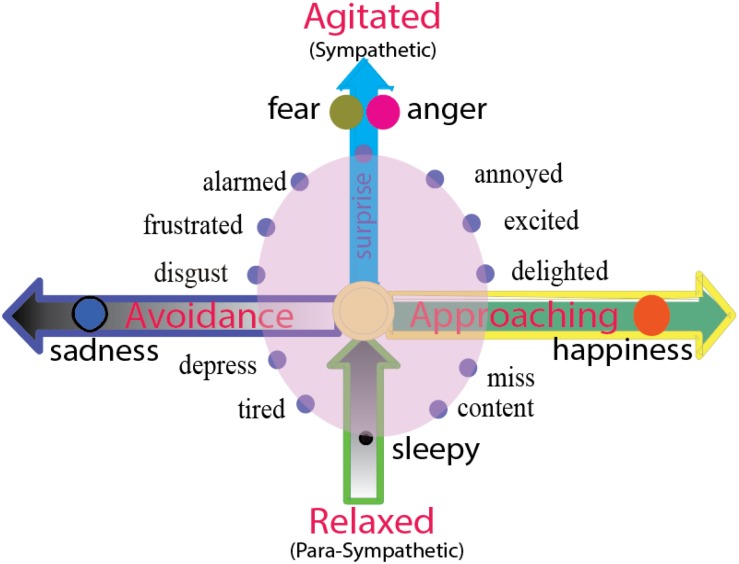
Two dimensions of emotion reflect the directions of behaviors or agitation of autonomous nervous system. The horizontal dimension represents the direction of the behavior, including the approaching/avoidance of the behavior. The emotion happiness and sadness and their behaviors prey or fleeing are on the opposite directions, happiness or joy induce approaching behavior, and sadness and disgust induce avoidance behavior. The vertical dimension represents the energy of the action (agitated or rest). Fear and anger or fight or flight might have same level of agitation, but they are twin emotions, standing back-to-back on the top of vertical axis, facing opposite direction (approaching or avoidance). The zero point of the vertical dimension means normal waking state, and negative axis means sleepy or tired, or the vertical dimensions above zero means sympathetic nervous system, and the dimensions below zero means para-sympathetic nervous system.

*Emotion is an innate state, it is a tendency of behavior*. Emotions carry behavioral intentions, and the readiness to act in certain ways (Roseman, 1984). The two independent dimensions not only decide two important features of emotions (hedonic value and arousal value), they can also decide the directions of behaviors that they will induce: approach and avoidance direction, and the strength of behavior (LeDoux, 1998; Ledoux and Brown, 2017) (Figure 3). Here, basic emotion induced behaviors can also be arranged in a two-dimension coordinate plane (Figure 3), with the horizontal dimension representing the direction of the behavior, including the approach/avoidance of the behavior, and the vertical dimension shows the agitation of the behavior. Thus, *the locations of the emotions on the dimensions can also be decided by the behaviors that they will induce*. “Fear and anger” induced “fight or flight” but these are different in the direction of the actions: Fear is in the negative direction (Certel et al., 2010), while fight is in the positive direction. Therefore, with happiness and sadness induced behaviors that are characteristic of prey or fleeing: Prey is in the positive direction while fleeing in the negative direction.

### Vertical Dimension-Agitation of Behaviors

While the horizontal dimension shows the direction of the behavior, the vertical dimension shows the agitation of the behavior, or the activities of autonomous nervous systems. The location above zero point means the activity of sympathetic nervous system, while the location below zero means the activity of the parasympathetic nervous system. “Fear and anger” induced “fight or flight” reflects the highest agitation of the sympathetic nervous system. The zero point means the normal waking state, and the negative axis means lethargy or sleepy. Freezing is in the special location “0” on the two-dimension coordinate plane (Figure 3). The fear and anger might have similar agitation, but they are different in the direction of the actions, fear is in the negative valence (Certel et al., 2010), while fight is in the positive direction. The vertical dimension represents the energy of the action (agitated or rest), so the fear and anger have the same volume of arousal.

### Fear and Anger Are Twin Emotions

**FIGURE 4:**
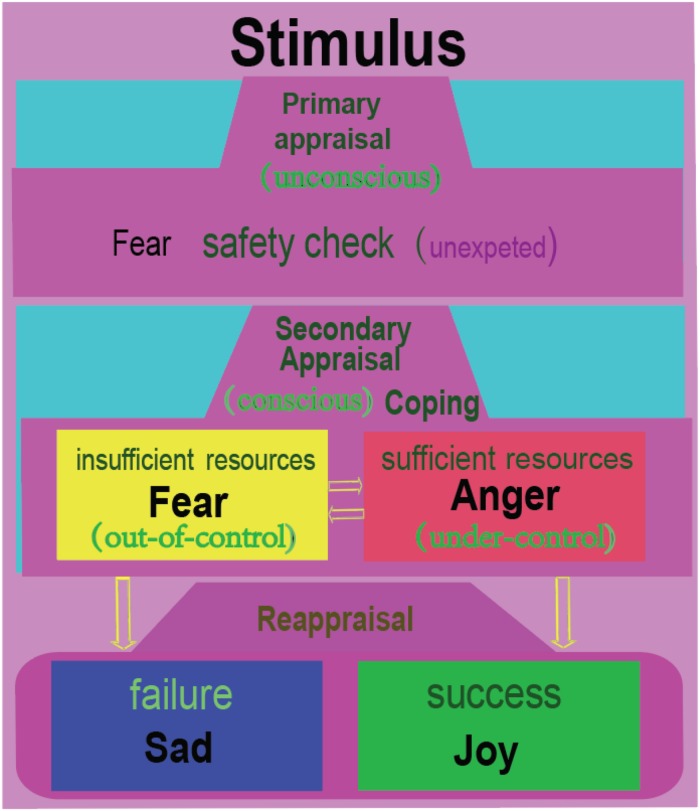
Fear and anger are twin emotions. At a surprise or uncertain situation, we humans usually have a safety check with the situation, which is unconscious, similar to Lazarus’s primary appraisal. Then the individual will consciously compare the situation with his own ability to see if he can cope with the situation. If the individual feels he has insufficient resources, he would flight away; or he will be angry and fight. Fear and anger occur in a tandem, with fear occurring first, then anger coming immediately afterward. Afterward, the individual will reflect upon the situation, which might be called reappraisal. If he coped successfully with the situation, he will be happy; or he will be sad. Therefore, we humans have four basic emotions. Just like Izard proposed: people need the emotion fear to explain flight for safety; anger to explain tendency to cope with the unexpected situation; joy or happiness to express the pride of achievement, and sadness to express the acceptance of failure (Izard, 2007).

Appraisal theory proposed that emotions are induced by the appraisal of a stimulus. Lazarus suggested that after meeting with a stimulus, the individual would first have an automatic, unconscious, and fast activating evaluation of the stimulus (Figure 4). Lazarus proposed that the primary appraisal is an unconscious judgment about the potential threat to an individual from a stressor (Lazarus, 1999). If the stimulus induces a threat, then the secondary appraisal will be induced. The secondary appraisal is conscious and concerned with coping (Lazarus, 1999; Zheng et al., 2016). Ledox also propose that the threat usually first induces fast unconscious fearful emotions, then induces angry emotions through cognitive comparisons (LeDoux, 1996). Thus, fear is related with uncertainty about the situation; and anger is related with trying to control the situation (Moons et al., 2010; Wang et al., 2017, 2018). The coping methods can be angry fight (having sufficient resources) or fearful flight (having insufficient resources). Therefore, we propose that fear can induce anger, while anger is the vent of fear (Gu et al., 2016; Zheng et al., 2016). Both fear and anger can aid survival by influencing an organism to either flight or fight for survival (LeDoux, 1998; Ledoux and Brown, 2017). Fear and anger are activated by norepinephrine systems, and they expressed similar sympathetic nervous activation (LeDoux, 1998; Zheng et al., 2016; Ledoux and Brown, 2017).

After coping with the stressful situation with direct actions, Lazarus suggested a kind of cognitive reappraisal processes: positive emotions will be induced after successfully coping with the situation, which can be named eudaimonic happiness (Berridge and Kringelbach, 2011); or negative emotions will be induced at failure to cope with the situation, which can be named sadness (Aldwin, 1994; Lazarus, 1999). The reappraisal can also be affected by cognition, therefore, positive reappraisal can induce better moods after stressful situations (Gross, 2002; Ochsner et al., 2002; Troy et al., 2010; Wang et al., 2017). The term “eudaimonia” is related to subjective feelings that an individual can feel when he is engaged in activities that are related to his skills or abilities contributing to his personal ambitions and life goals (Ryff, 2014, 2018). So when something unexpected occurs, the individual will first evaluate its threat (fear/anger) and next evaluate its hedonic value (happy/sad) (Gu et al., 2015, 2018). Indeed, basic emotions are transient and interchangeable, which makes it difficult to be differentiated with fMRI studies, which has temporal limitations (Kringelbach, 2005).

### Four Basic Emotions on Two Dimensions

Many psychologists have proposed that disgust and surprise are basic emotions, such as Ekman, Plutchik. However, disgust is kind of strong dislike, or disgust has something to do with the arousal. This means disgust does not locate on the hedonic axis, instead it is biased toward the arousal dimension. Surprise was named as one basic emotion by many psychologists, it actually might be a core affect instead of an emotion. Surprise seems like a property of a stimulus, instead of an emotion. Therefore, surprise might be a better name for the vertical dimension, or surprise should be a core affect instead of a basic emotion.

All in all, we humans have four basic emotions: Happiness and sadness represent the horizontal dimension, reflecting the hedonic value of the stimulus (Figure 2); while fear and anger are on the vertical axis, depending on the surprising way the stimulus happens. Just like what Izard proposed: People need the emotion fear to explain flight for safety; anger to explain tendency to cope with the unexpected situation; joy or happiness to express the pride of achievement, and sadness to express the acceptance of failure (Izard, 2007). With this in mind, it might be easier to understand the fMRI results, which found that some basic emotions induced similar brain activities, for fear, anger and surprise located on the same location of the vertical dimension. This might be the reason that fMRI studies found that activation of limbic and paralimbic brain regions are not specific to special basic emotions (for example, amygdala is not for fear, anger, or surprise), instead they are related to the core affects (Kragel and LaBar, 2016); or the orbitofrontal cortex responds not only to specific instances of positive emotions, instead they respond to hedonic value, and can be named as “salience network”. In addition, the analyses found that subjective rating of valence predicted the similar responses in mesolimbic dopaminergic systems (Kamitani and Tong, 2005; Lindquist et al., 2012).

## Conclusion

The basic emotion theory hypothesizes that basic emotion (and the emotions that are derived from this basic emotion) is produced by the activity of a defined brain locus or an anatomically defined network (Farinelli et al., 2015). Recently, this approach has incorporated efforts to map basic emotions to brain networks that comprise basic emotions (fear, anger, happiness and sadness) (Cowen and Keltner, 2018; Selvaraj et al., 2018). However, fMRI studies cannot achieve consistent imaging data for specific brain areas for a specific emotion. Thus, many researchers suggested an alternative way to explain it: the psychological constructionist model of emotion, hoping that we can get consistent brain activities for core affects in fMRI experiments, or there are specific neural structures that are involved in arousal and hedonic value. Even though hedonic value can achieve consistent activities in the anterior insular, OFC, caudate, thalamus and anterior cingulate, arousal values still demonstrate some trouble in the fMRI studies (Lewis et al., 2007; Kuppens et al., 2013; Lindquist et al., 2013a).

Here we have reviewed brain imaging and neuropsychological data, and addressed this question with an integrative model. In this model, we proposed that basic emotions are not contrary to the dimensional studies of emotions (core affect). Instead, basic emotions can be explained more clearly with the dimensional studies. We also give a criteria for basic emotions: Basic emotion should locate on the axis in the dimensions of emotion, and only represent one typical core affect (arousal or valence). Therefore, we proposed four basic emotions: joy-on positive axis of hedonic dimension, sadness-on negative axis of hedonic dimension, fear and anger which are twin emotions and locate on the top of vertical dimensions. This new model for basic emotions and the construction model of emotions is promised to improve and reformulate neurobiological models of basic emotions.

## Author Contributions

SG and FW designed the manuscript. SG, FW, EW, YT, and JH wrote the manuscript. CC revised the manuscript.

## Conflict of Interest Statement

The authors declare that the research was conducted in the absence of any commercial or financial relationships that could be construed as a potential conflict of interest.
